# Supplementary material for: A Novel CreA-Mediated Regulation Mechanism of Cellulase Expression in the Thermophilic Fungus Humicola insolens
Source: Int J Mol Sci. 2019 Jul 28;20(15):3693. doi: 10.3390/ijms20153693 (PMC6696435; doi:10.3390/ijms20153693)
Supplement: Supplementary file 1 [file ijms-20-03693-s001.pdf]

**Supplementary Materials**

**Table S1.** Primers used in this study.

| Primer | Sequence (5′-3′) [a] |
|---|---|
| *creA*LS | TAGGCGCGCAAGGATCCTCTTTGATGAGGGCGAAATGG |
| *creA*LA | CACTAGTCGGGGGATCCTCTCAGCAGGTCCGAGAAGTGT |
| *creA*RS | TTAATTAATTGACCGACATCATAAACCG |
| *creA*RA | TTAATTAACAACCATCCAGGCATCTAA |
| *creA*S | TTAATTAAACATCCCAAGACGCAATCC |
| *creA*A | TTAATTAAGCAAGACCCAGGTGACAAA |
| *creA*IS | GGCTGGAGCACCAAACACG |
| *creA*IA | GCCGCGACAGATGGTAAGC |
| *neo*qF | TTGGGTGGAGAGGCTATTCG |
| *neo*qR | CTTCCCGCTTCAGTGACAAC |
| *acti*qF | GAGTTGAAGGTGGTGACGTG |
| *acti*qR | AGCGTGAAATTGTCCGTGAC |
| *cel6A*qF | ATTCCTCAGATCACCGACCC |
| *cel6A*qR | CGGTCAGGAAGGTCGTAAAC |
| *cel6B*qF | CCACATCCACCTCTACCTGG |
| *cel6B*qR | AGGGGTTGTAGTTGGAGACG |
| *cel7A*qF | GTCCAGGCTTCCATCACTCT |
| *cel7A*qR | ATAGGTGCTGGTGTAGTCGG |
| *cel7B*qF | TCTGTACCTGTCCGAGATGC |
| *cel7B*qR | GTACAGGCCCTTCTTGTTGC |
| *bgl3A*qF | GATTCAACATCACCGAGGCC |
| *bgl3A*qR | CGCTCATAACGAAACCCTGG |
| *bgl3B*qF | TGGCTCAGTCACATCTCAGG |
| *bgl3B*qR | GCCCAAACTTGTACCAGGTG |
| *bgl3C*qF | AGGTTTCTCTCCACAGCTCC |
| *bgl3C*qR | ACGATGGTGTTCTTGTTGGC |
| *xynA*qF | TCTACAAGGTTCTCGGCGAG |
| *xynA*qR | CTTGAGGAGCTTGACGATGC |
| *xynB*qF | CAGTTTACCTTCGGCTCTGC |
| *xynB*qR | CATTGACGACATCCCAGTGG |
| *xynC*qF | TCAACAAGTGGGTTTCGCAG |
| *xynC*qR | CTTCTGGACGTTGAGGCAAG |

[a] The underlined nucleotide sequences indicate restriction enzyme sites.

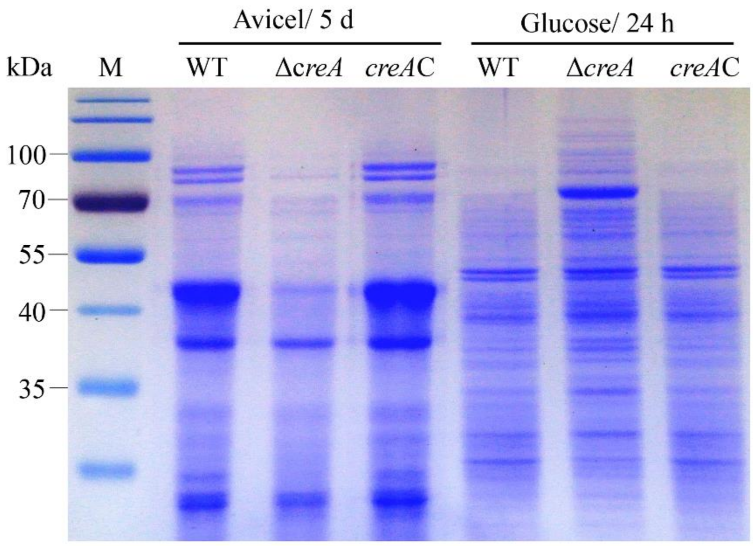


**Figure S1.** Extracellular protein profiles of the H. insolens creA disruption mutant, creA complementary strains, and the WT. Sodium dodecyl sulfate–polyacrylamide gel electrophoresis (SDS–PAGE) of the extracellular protein profiles of all strains after fermentation with Avicel for 5 days or glucose for 24 h. M, PageRuler pre-stained protein ladder.
